# Phase 1 pharmacokinetic study of MK-0646 (dalotuzumab), an anti-insulin-like growth factor-1 receptor monoclonal antibody, in combination with cetuximab and irinotecan in Japanese patients with advanced colorectal cancer

**DOI:** 10.1007/s00280-013-2240-8

**Published:** 2013-08-07

**Authors:** Toshihiko Doi, Kei Muro, Takayuki Yoshino, Nozomu Fuse, Takashi Ura, Daisuke Takahari, Hwa-ping Feng, Takashi Shimamoto, Kazuo Noguchi, Atsushi Ohtsu

**Affiliations:** 1 National Cancer Center Hospital East, 6-5-1, Kashiwanoha, Kashiwa, Chiba 277-8577 Japan; 2 Department of Clinical Oncology, Aichi Cancer Center Hospital, 1-1 Kanokoden, Chikusa-ku, Nagoya, Aichi 464-8681 Japan; 3 Merck & Co., Inc., Whitehouse Station, NJ USA; 4 MSD K.K., Kitanomaru Square, 1-13-12, Kudan-kita, Chiyoda-ku, Tokyo 102-8667 Japan

**Keywords:** Colorectal cancer, MK-0646, Anti-IGF-1R antibody, Pharmacokinetic interactions, Phase I study

## Abstract

**Purpose:**

The safety, tolerability, and pharmacokinetic (PK) interactions of MK-0646 in combination with cetuximab and irinotecan were investigated in Japanese patients with advanced colorectal cancer.

**Methods:**

Twenty patients were treated in the following study arms in combination with cetuximab and irinotecan: A [MK-0646 (10 mg/kg) weekly starting on Day 22], B [MK-0646 (15 mg/kg) on Day 8, followed by 7.5 mg/kg every 2 weeks], or C [MK-0646 (10 mg/kg) on Day 1 and weekly starting on Day 22]. Dose limiting toxicities (DLTs) were evaluated during a prespecified 4-week period in arms A and B. Full PK sampling was performed to evaluate the PK interactions.

**Results:**

One of the 6 evaluable patients in arm A developed a DLT (grade 3 hyperglycemia); no DLTs occurred in the 6 patients in arm B. Common treatment-related adverse events included leukopenia, neutropenia, dermatitis acneiform, paronychia, nausea, stomatitis, diarrhea, and decreased appetite. The co-administration of cetuximab and irinotecan with MK-0646 increased the MK-0646 AUC_0–168h_ by 25 %, with MK-0646 accumulation from the previous dose contributing to the observed increase. The co-administration of MK-0646 with cetuximab and irinotecan did not affect the PK of cetuximab and irinotecan, but reduced the *C*_max_ (from 16.8 to 13.0 ng/mL) and the AUC_0–24h_ (by 13 %) of SN-38, the active metabolite of irinotecan.

**Conclusions:**

The triple combination of MK-0646, cetuximab, and irinotecan was well tolerated in Japanese patients with advanced colorectal cancer. These results indicate a minimal potential for PK interactions between MK-0646 and cetuximab and between MK-0646 and irinotecan/SN-38.

## Introduction

Colorectal cancer is the third most common cause of cancer-related death in both men and women worldwide. For patients with metastatic colorectal cancer, the standard of care using a fluoropyrimidine, irinotecan, oxaliplatin, and bevacizumab (in combination or sequentially) results in a median survival period of 18–21 months [1–4]. However, once these standard drugs have failed, further options are limited. Such patients with progressive metastatic disease, despite having received currently available first- and second-line chemotherapies, and who exhibit the tumor-specific expression of the epidermal growth factor receptor (EGFR) are eligible to receive third-line treatment with irinotecan and cetuximab. Cetuximab is a monoclonal antibody that specifically blocks EGFR, a member of the ErbB family of receptors [5]. EGFR is overexpressed in up to 80 % of colorectal cancers and is associated with a poor survival outcome [6–8]. Despite treatment with cetuximab, the prognosis for patients in this population remains poor, with a response rate of 22.9 %, a median time to progression of 4.1 months, and a median overall survival period of 8.6 months [9].

MK-0646 (dalotuzumab) is a humanized IgG1 kappa antibody targeting insulin-like growth factor receptor type 1 (IGF-1R). Signaling through IGF-1R mediates cell growth and proliferation as well as resistance to apoptosis in all major solid tumors, including colorectal cancer [10]. MK-0646 has two possible mechanisms of action: (1) the inhibition of IGF-1-mediated cell signaling, and (2) antibody-dependent, cell-mediated cytotoxicity (ADCC). A preclinical study suggested a synergistic effect on tumor growth inhibition when combined with either a chemotherapeutic agent or an anti-EGFR antibody [10]. Additionally, emerging evidence suggests crosstalk between the EGFR and IGF-1R signaling pathways [11]. Hence, the concurrent inhibition of IGF-IR and EGFR provides a logical rationale for combining anti-IGF-IR and anti-EGFR strategies in the treatment of cancer.

A phase I study of single-agent MK-0646 was conducted in patients with advanced solid tumors [12]. MK-0646 was generally well tolerated and exhibited dose-proportional pharmacokinetics (PKs). The safety and tolerability of a triple combination of MK-0646, cetuximab, and irinotecan was evaluated in an open-labeled safety run-in prior to commencing a blinded randomized phase II/III study [13]. The results suggested that the triple combination of MK-0646, cetuximab, and irinotecan was tolerable, with no overlapping toxicities highlighted in non-Japanese patients with metastatic colorectal cancer.

In the present study, the safety, tolerability, and PK of MK-0646 in combination with cetuximab and irinotecan in Japanese patients with advanced colorectal cancer were investigated. The potential for PK interactions between MK-0646 and cetuximab and between MK-0646 and irinotecan as well as SN-38, the active metabolite of MK-0646, was assessed. The tumor response to this triple combination was also evaluated as an exploratory objective.

## Materials and methods

### Patient eligibility

This study was conducted based on the Declaration of Helsinki and the Guidelines for the Clinical Evaluation Methods of Anti-Cancer Drugs in Japan (Japanese Ministry of Health, Labour, and Welfare notification, dated November 1, 2005). The study was approved by the institutional review board of each study site.

The main eligibility criteria were as follows: histologically (or cytologically) confirmed advanced colorectal cancer that had previously failed to respond to both irinotecan and oxaliplatin and had progressed on or within 3 months of the last therapy; a patient age of 20 years or older; an Eastern Cooperative Oncology Group performance status of 0 or 1; and adequate hematological, hepatic, and renal functions. The exclusion criteria included the use of chemotherapy, radiotherapy, or biological therapy within 4 weeks prior to enrollment; primary or unstable central nervous system metastasis; and symptomatic ascites or pleural effusion requiring treatment. All the patients provided informed consent, and the trial was conducted in accordance with current good clinical practice standards. This trial was registered at ClinicalTrials.gov as NCT00925015.

### Drug administration

In arm A, cetuximab at a dose of 400 mg/m^2^ was infused over 120 min as a loading dose on Day 1 of Cycle 1. On the following weeks, cetuximab at a dose of 250 mg/m^2^ was infused over 60 min once weekly as a maintenance dose. Irinotecan at a dose of 150 mg/m^2^ was infused over 90 min every other week. MK-0646 at a dose of 10 mg/kg was infused over 120 min once weekly starting on Day 22 of Cycle 1.

In arm B, cetuximab at a dose of 400 mg/m^2^ was infused over 120 min as a loading dose on Day 1 of Cycle 1. On subsequent weeks, cetuximab at a dose of 250 mg/m^2^ was infused over 60 min once weekly as a maintenance dose. Irinotecan was infused over 90 min according to the dosage and regimen that the patient had most recently received prior to enrollment in this study. MK-0646 was infused over 120 min once every other week starting on Day 8 of Cycle 1. The first infusion was a loading dose of 15 mg/kg, while all subsequent infusions consisted of a dose of 7.5 mg/kg.

In arm C, cetuximab at a dose of 400 mg/m^2^ was infused over 120 min as a loading dose on Day 8 of Cycle 1. On following weeks, cetuximab at a dose of 250 mg/m^2^ was infused over 60 min once weekly as a maintenance dose. Irinotecan was infused every other week over 90 min at a dose of 150 mg/m^2^ starting on Day 8 of Cycle 1. On Day 1 of Cycle 1, MK-0646 at a dose of 10 mg/kg was infused over 120 min. On Day 22 (following a 2-week rest-period) and thereafter, MK-0646 was infused at a dose of 10 mg/kg over 120 min once weekly.

The study treatments were continued until the patient exhibited disease progression or the occurrence of an unacceptable toxicity.

### Study design and evaluation

**Fig. 1:**
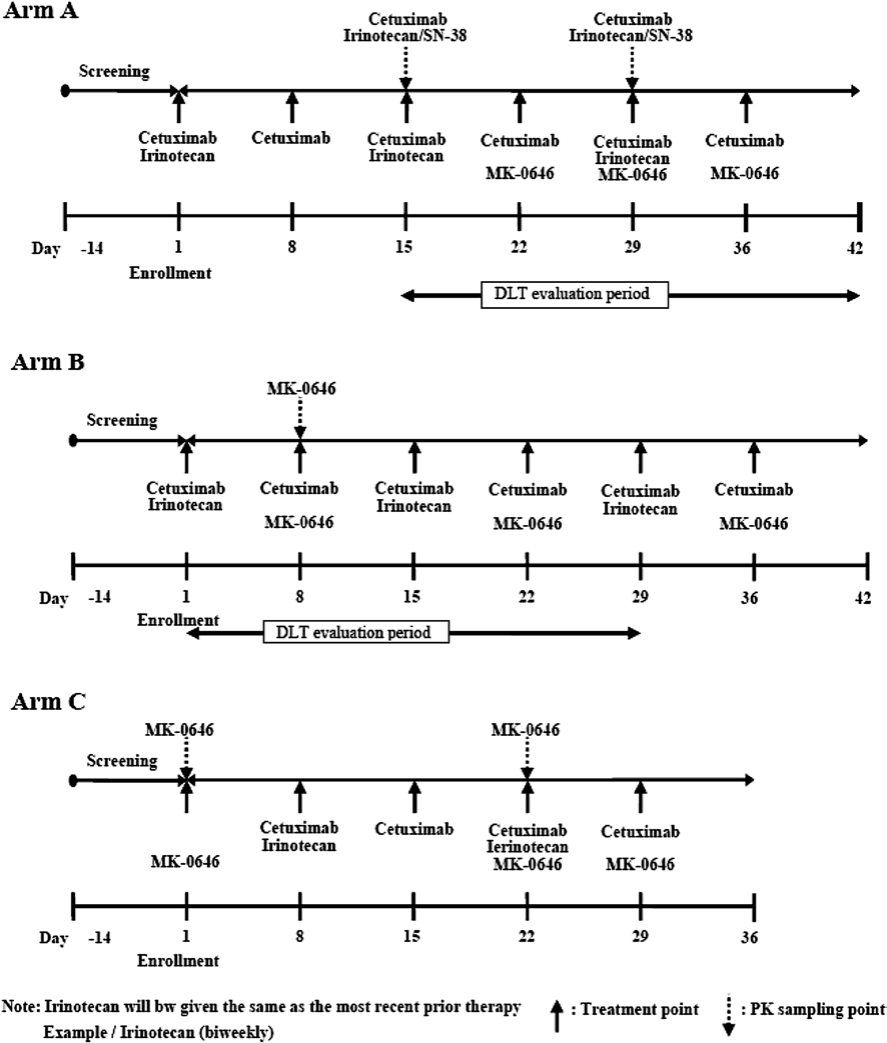
Study design

This study was an open-label, nonrandomized, multi-center phase I study of MK-0646 in patients with advanced colorectal cancer (Fig. 1). All eligible patients were treated in either arm A, B, or C. The DLT assessment for the weekly (10 mg/kg) and every other week regimen (an initial dose of 15 mg/kg followed by a maintenance dose of 7.5 mg/kg) for the triple combination of MK-0646, cetuximab, and irinotecan in Japanese patients was conducted in arms A and B, respectively. The prespecified 4-week period of Cycle 1 was defined as the DLT evaluation period (arm A: Day 15–Day 42, arm B: Day 1–Day 28). Patients were enrolled in arm B in parallel with those in arm A using a 3 + 3 design. In arms A and B, if ≤2 of the first three patients developed a DLT, then three additional patients were enrolled at the same dose level. If all of the first 3 patients developed a DLT, the enrollment of the additional 3 patients in the arm was canceled. Enrollment in this study was sequential, not randomized. If 2 or less of the 6 patients manifested a DLT during the 4 weeks of Cycle 1 in arms A and B, the dosing regimen was considered tolerable.

Adverse events were graded using the National Cancer Institute Common Terminology Criteria for Adverse Events, version 3.0. DLT was defined as grade 4 neutropenia lasting >5 days; grade 3 or 4 neutropenia with a fever >38.5 °C; grade 4 thrombocytopenia; or grade 3 or 4 nonhematological toxicity, except for inadequately treated diarrhea, nausea and vomiting, rash, hyperglycemia, and transient electrolyte abnormalities. Anemia was not considered to be a DLT; patients were allowed blood transfusions as needed. Infusion reactions and hypersensitivity reactions were not considered to be DLTs.

Additionally, the dosing schedule for arm A was designed to assess the effect of MK-0646 on the PK parameters of cetuximab, irinotecan, and its metabolite (SN-38). The dosing schedule for arm C was designed to assess the effect of cetuximab and irinotecan on the PK parameters of MK-0646.

The anti-tumor activity was evaluated at baseline and every 6 weeks according to RECIST, version 1.0.

### Pharmacokinetics

Blood samples for the measurement of the serum MK-0646 concentration were collected on Day 1 and Day 22 of Cycle 1 in arm C (pre-dose, end of infusion, and 0.5, 5.0, 8, 24, 48, 96, and 168 h after the start of infusion). The serum MK-0646 concentration was determined using a validated enzyme-linked immunosorbent assay (ELISA) with colorimetric detection at Tandem Labs. MK-0646 was captured with immobilized recombinant human IGF-1R and was detected using biotinylated mouse antihuman IgG1Fc, developed by the addition of streptavidin-HRP and TMB substrate. The calibration range of the assay was 20–2,000 ng/mL in 100 % serum, with an LLOQ of 20 ng/mL.

Blood samples for the measurement of the serum cetuximab concentration were obtained on Day 15 and Day 29 of Cycle 1 in arm A (pre-dose and 2.0, 5.0, 8.0, 24, 48, 96, and 168 h after the start of infusion). The serum cetuximab concentration was determined using a validated electrochemiluminescence (ECL) assay at Tandem Labs. Cetuximab was captured using biotinylated anti-cetuximab anti-idiotype antibody and was detected with TAG-labeled anti-cetuximab anti-idiotype antibody. The calibration range of the assay was 3.91–250 ng/mL in 50 % serum, with an LLOQ of 7.8 ng/mL in 100 % serum.

Blood samples for the measurement of the plasma irinotecan concentrations were obtained on Day 15 and Day 29 of Cycle 1 in arm A (pre-dose and 1.0, 5.0, 8.0, 24, and 48 h following the completion of the infusion). The plasma irinotecan and SN-38 concentrations were determined using validated LC-MS/MS methods at CEDRA Clinical Research LCC. The calibration range of the assays was 2–1,000 ng/mL for irinotecan and 1–500 ng/mL for SN-38.

The MK-0646, cetuximab, irinotecan, and SN-38 PK parameters were determined using a noncompartment analysis and the serum or plasma concentrations of each analyte and the actual sampling times relative to the actual dose times. The AUC was natural log-transformed and analyzed using a linear mixed effects model, with fixed-effect terms for treatment (with or without co-administered drugs). An unstructured covariance matrix was used to allow for unequal treatment variances and to model the correlation between the two treatment measurements within each subject via the REPEATED statement in SAS PROC MIXED. Kenward and Roger’s method was used to calculate the denominator degrees of freedom for the fixed effects (DDFM = KR). Exponentiating the least-squares means (mean differences) and the lower and upper limits of these confidence intervals yielded estimates for the population geometric means (population geometric mean ratios) and confidence intervals for the geometric means (geometric mean ratios) on the original scale. A 95 % confidence interval (CI) was constructed for the geometric means of the AUCs for the treatment arms. To assess the effect of drug interactions, 90 % CI was constructed for the geometric mean ratios (GMRs) (with co-administered drugs vs. administration alone) of the AUC.

### Immunogenicity

A sandwich format ELISA assay was developed for detecting the incidence of human anti-humanized antibodies (HAHA) in response to MK-0646 therapy. The presence of HAHA was measured by analyzing sera collected from patients prior to the administration of the first dose of study medication, every 6 weeks during the study period, and 4, 8, and 12 weeks posttreatment after the last dose of study medication.

## Results

### Patient characteristics

Twenty Japanese patients with advanced colorectal cancer were enrolled and were evaluated in this study. The baseline characteristics of the patients are summarized in Table 1. The age range was 51–71 years (median 61.5 years). Eight patients had colon cancer, and 12 patients had rectal cancer. The KRAS status was wild-type in 10 patients, mutant-type in 7 patients, and unknown in 3 patients. The median number of prior chemotherapy regimens was 3.0. Two patients enrolled in arm A were excluded from the DLT evaluation. One patient had grade 3 febrile neutropenia before the first administration of MK-0646, and this patient only received cetuximab and irinotecan. The other patient had grade 3 skin toxicity before the start of the DLT evaluation period. Thus, two additional patients were enrolled in arm A (total of 8 patients). Six patients were treated in arms B and C, respectively. The median number of treatment cycles (1 cycle: 6 weeks) was 2.0 (range 1.0–3.0) in arm B, 2.0 (range 1.0–7.0) in arm B, and 2.5 (range 1.0–5.0) in arm C. The patients discontinued the study medication because of adverse events (*n* = 4), their physician’s decision (*n* = 1), progressive disease (*n* = 14), or the withdrawal of consent (*n* = 1).

**Table 1:** Baseline characteristics of the patients

Characteristics	Arm A (*n* = 8)	Arm B (*n* = 6)	Arm C (*n* = 6)	All patients (*n* = 20)
Age (years)				
Median	64.0	60.5	57.0	61.5
Range	52–-68	53–62	51–71	51–71
Sex, *n* (%)				
Male	5	5	5	15
Female	3	1	1	5
Weight (kg)				
Median	55.6	51.8	67.3	57.2
Range	47.0–70.0	37.0–68.0	44.0–83.0	37.0–83.0
ECOG performance status, *n* (%)				
0	4	1	4	9
1	4	5	2	11
Primary tumor, *n* (%)				
Colon cancer	3	3	2	8
Rectal cancer	5	3	4	12
Stage of disease, *n* (%)				
IV	8	6	6	20
KRAS status				
Wild	4	3	3	10
Mutant	4	2	1	7
Unknown	0	1	2	3
Median no. of prior chemotherapy	2.5	4.0	2.0	3.0
Range	2–3	2–6	2–5	2–6

### Safety and tolerability

Of the 6 patients who were evaluated for DLTs in arm A, none of the patients developed a DLT. Of the 6 patients who were evaluated for DLTs in arm B, one patient developed a DLT (grade 3 hyperglycemia). The time until the onset of the DLT after the administration of the study medication was 15 days, and the DLT resolved after the study medications were interrupted and treatment with an anti-hyperglycemic agent (pioglitazone) was initiated. The common drug-related adverse events reported for all the treatment cycles in all the arms are summarized in Table 2. The most common hematological adverse events related to the study medications (MK-0646 and/or cetuximab and/or irinotecan) included leukopenia (15/20; 75.0 %) and neutropenia (14/20; 70.0 %). The most common nonhematological adverse events included dermatitis acneiform (13/20; 65.0 %), paronychia (13/20; 65.0 %), nausea (12/20; 60.0 %), stomatitis (11/20; 55.0 %), diarrhea (11/20; 55.0 %), and decreased appetite (10/20; 50.0 %).

**Table 2:** Common adverse events related to study medications

	Arm A (*n* = 8)		Arm B (*n* = 6)		Arm C (*n* = 6)		All patients (*n* = 20)	
	All grades	Grades 3–4	All grades	Grades 3–4	All grades	Grades 3–4	All grades	Grades 3–4
Blood and lymphatic System disorders								
Leukopenia	7	4	2	1	6	2	15	7
Neutropenia	7	4	2	1	5	2	14	7
Lymphopenia	4	2	0	0	3	1	7	3
Thrombocytopenia	3	0	1	0	1	0	5	0
Anemia	2	0	0	0	2	0	4	0
Skin and subcutaneous tissue disorders								
Dermatitis acneiform	7	3	1	0	5	2	13	5
Dry skin	2	0	3	0	0	0	5	0
Acne	0	0	4	0	0	0	4	0
Alopecia	2	–	1	–	1	–	4	–
Pruritus	2	0	2	0	0	0	4	0
Infections and infestations								
Paronychia	6	2	2	2	5	2	13	6
Gastrointestinal disorders								
Nausea	5	0	4	0	3	0	12	0
Stomatitis	5	0	3	0	3	0	11	0
Diarrhea	3	0	5	0	3	0	11	0
Vomiting	1	0	2	0	3	0	6	0
Constipation	1	0	2	0	2	0	5	0
Metabolism and nutrition disorder								
Decreased appetite	4	0	4	1	2	0	10	1
Hyperglycemia	2	0	3	1	2	0	7	1
Hypomagnesemia	2	0	2	1	3	1	7	2
Hypoalbuminemia	3	0	1	0	1	0	5	0
General disorders								
Fatigue	4	1	4	0	1	0	9	1
Pyrexia	1	0	1	0	2	0	4	0
Investigations								
Weight decreased	3	0	1	0	1	0	5	0

All grades of adverse events reported in 4 or more patients are listed

### Pharmacokinetic evaluation

#### MK-0646

**Fig. 2:**
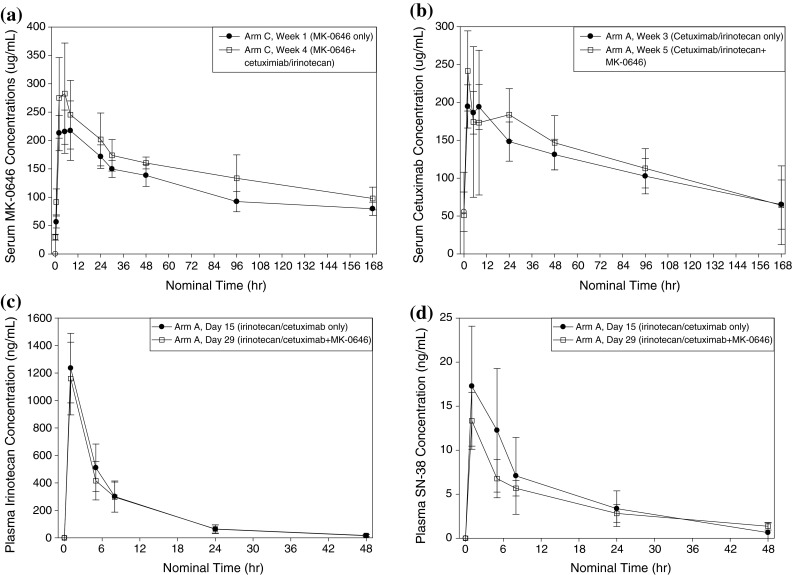
Mean concentration versus time profiles for MK-0646 (**a**), cetuximab (**b**), irinotecan (**c**), and SN-38 (**d**)

The mean serum concentration profiles for MK-0646 after a 2-h IV infusion of 10 mg/kg of MK-0646 (arm C) are shown in Fig. 2. Descriptive statistics for the PK parameters are given in Table 3. The mean MK-0646 serum concentration after the co-administration of MK-0646 with cetuximab/irinotecan during Week 4 (Day 22) was higher than that after MK-0646 administration alone during Week 1 (Day 1). The arithmetic mean pre-dose MK-0646 serum concentration during Week 4 was 29.7 μg/mL, indicating the accumulation of MK-0646. The median time to reach *C*_max_ (*T*_max_) was 5 h post-dose for the MK-0646 alone treatment and 3.5 h post-dose for the MK-0646 + cetuximab/irinotecan treatment. The exposure to MK-0646 upon co-administration with cetuximab and irinotecan during Week 4 was slightly higher than that when administered alone during Week 1 (the geometric mean of the *C*_max_ increased from 247.6 to 311.9 μg/mL; the geometric mean of the AUC_0–168h_ increased from 19.6 to 24.5 mg h/mL). The GMR and the 90 % CI of the AUC_0–168h_ for the two treatments (MK-0646 + cetuximab/irinotecan vs. MK-0646 alone) were 1.25 and (1.15, 1.35).

**Table 3:** Pharmacokinetic parameters for MK-0646 following the administration of MK-0646 alone (Day 1) or in combination with cetuximab/irinotecan (Day 22)

PK parameters	Day 1 (*n* = 6) MK-0646	Day 22 (*n* = 6) Triple combination
*T*_max_ (h)^a^	5.0 (2.0–8.0)	3.5 (2.0–24.0)
*C*_eoi_ (μg/mL)^b^	211.2 (14.3)	267.3 (27.1)
*C*_max_ (μg/mL)^b^	247.6 (14.4)	311.9 (21.3)
*t*_1/2_ (h)^b^	131.4 (21.5)	141.4 (32.1)
CL (mL/min/kg)^b^	0.0049 (21.9)	0.0038 (33.7)
Vss (L/kg)^b^	0.0558 (11.1)	0.0459 (14.2)
AUC_0–24h_ (mg h/mL)^b^	4.56 (11.7)	5.39 (18.8)
AUC_0–168h_ (mg h/mL)^b^	19.6 (13.1)	24.5 (19.0)

^a^Median (range)

^b^Geometric mean (coefficient of variation)

#### Cetuximab

The mean serum concentration profiles for cetuximab after a 2-h IV infusion at 250 mg/m^2^ are shown in Fig. 2. The PK parameters are given in Table 4. The co-administration of MK-0646 with cetuximab/irinotecan produced a higher mean peak cetuximab concentration (geometric mean of the *C*_max_ value of 236.5 vs. 204.0 μg/mL without MK-0646 co-administration), which was reached earlier (median *T*_max_ value of 2.0 vs. 7.9 h without MK-0646 co-administration). The AUC of cetuximab was not altered by the co-administration of MK-0646. The GMR of the cetuximab AUC_0–168h_ (cetuximab + irinotecan + MK-0646/cetuximab + irinotecan) was 1.07, with a 90 % CI of (0.94, 1.21), which was within the (0.80, 1.25) bioequivalence bounds.

**Table 4:** Pharmacokinetic parameters for cetuximab, irinotecan, and SN-35 following the administration of cetuximab/irinotecan alone (Day 15) or in combination with MK-0646 (Day 29)

PK parameters	Cetuximab		Irinotecan		SN-38	
	Day 15 (*n* = 7) Cetuximab/irinotecan	Day 29 (*n* = 6) Triple combination	Day 15 (*n* = 7) Cetuximab/irinotecan	Day 29 (*n* = 6) Triple combination	Day 15 (*n* = 7) Cetuximab/irinotecan	Day 29 (*n* = 6) Triple combination
*T*_max_ (h)^a^	7.9 (2.0–8.0)	2.0 (2.0–2.1)	1.0 (0.97–1.0)	1.0 (1.0–1.1)	1.0 (0.97–5.0)	1.0 (1.0–1.1)
*C*_max_ (μg/mL)^b^	204.0 (15.0)	236.5 (23.0)	1.21 (22.8)	1.13 (25.6)	0.0168 (33.8)	0.0130 (23.9)
*t*_1/2_ (h)^b^	129.4 (26.0)	131.9 (34.5)	8.95 (9.13)	9.67 (12.7)	12.8 (32.0)	19.4 (12.6)
AUC_0–24h_ (μg h/mL)^b^	4,020 (14.5)	4,120 (26.1)	7.19 (33.6)	6.64 (31.9)	0.157 (51.1)	0.126 (21.3)
AUC_0–168h_ (μg h/mL)^b^	18,600 (20.9)	20,100 (28.6)	–	–	–	–
CL (mL/h/m^2^)^b^	7.81 (32.8)	6.82 (40.5)	12,300 (35.7)	13,100 (34.3)	–	–
Vss (L/m^2^)^b^	1.45 (10.0)	1.32 (17.8)	117 (27.5)	137 (24.9)	–	–

^a^Median (range)

^b^Geometric mean (coefficient of variation)

#### Irinotecan/SN-38

The mean plasma concentration profiles of irinotecan and SN-38 after a 30-min IV infusion at 150 mg/m^2^ are shown in Fig. 2. The PK parameters are given in Table 4. The mean irinotecan PK parameters with and without MK-0646 co-administration were comparable. The GMR of the irinotecan AUC_0–24h_ was 0.95, with a 90 % CI of (0.90, 1.01), which was within the bioequivalence bounds (0.80, 1.25). The co-administration of MK-0646 with cetuximab/irinotecan reduced the peak SN-38 concentration (*C*_max_ value of 13.0 vs. 16.8 ng/mL without MK-0646 co-administration). The median *T*_max_ was the same for both treatments (1.0 h). The SN-38 AUC was reduced after co-administration with MK-0646 (126 vs. 157 ng h/mL), and the elimination half-life was increased upon the co-administration of MK-0646 (19.4 vs. 12.8 h). The SN-38 AUC_0–24h_ GMR was 0.87, with a 90 % CI of (0.74, 1.01). The lower 90 % CI bound was outside the bioequivalence bounds (0.80, 1.25).

### Antitumor activity

As an exploratory objective, the tumor response to the triple combination of MK-0646, cetuximab, and irinotecan was evaluated according to the RESICT guidelines. Of the 20 patients whose response could be evaluated, 3 patients achieved a partial response (3/20; 15.0 %) as the best response: one patient with colon cancer in arm B (the time to response was 37 days) and two patients with rectal cancer in arms B and C (the times to response were 33 and 74 days, respectively). The KRAS status of the 3 responders was wild-type in 1 patient, mutant-type in 1 patient, and unknown in 1 patient. The duration of the response was 250 days for the patient with rectal cancer in arm B, but this parameter was not calculated for the other two patients because these patients discontinued the treatment because of adverse events.

### Immunogenicity evaluation

No HAHA was detected in the serum samples obtained throughout the study.

## Discussion

The primary objective of this study was to evaluate the safety and tolerability of MK-0646 in combination with cetuximab irinotecan in Japanese patients with advanced colorectal cancer. Of the six patients who were evaluated for DLTs in arm A (MK-0646, 10 mg/kg weekly), none of the patients developed a DLT. Of the six patients who were evaluated for DLTs in arm B (MK-0646, 15 mg/kg as a loading dose followed by 7.5 mg/kg every 2 weeks), one patient developed a DLT (grade 3 hyperglycemia). The grade 3 hyperglycemia reported in this study was adequately controlled with the interruption of MK-0646 treatment and the administration of an oral anti-hyperglycemia agent; the patient was then able to continue the study medications with a reduced dose of MK-0646. In this study, hyperglycemia was reported in 7 (6 with grade 1–2, 1 with grade 3) of the 20 patients treated with the triple combination. Hyperglycemia is recognized as an adverse event of treatment with anti-IGF-1R antibodies [14]. In a previous study examining single-agent MK-0646 in non-Japanese patients, hyperglycemia that was thought to be treatment related was reported in 15 (14 with grade 1–2, 1 with grade 3) of the 80 patients treated with MK-0646 [11]. The incidence of hyperglycemia reported for the triple combination therapy was higher than that for the MK-0646 monotherapy, but the small sample size of the present study prevents any definite conclusions from being made. The common treatment-related adverse events observed in this study included leukopenia, neutropenia, dermatitis acneiform, paronychia, nausea, stomatitis, diarrhea, and decreased appetite. These adverse events have also been reported for the single-agent uses of MK-0646, cetuximab, and irinotecan. No significant increases in grade 3 or 4 adverse events were observed for the combination treatment, compared with monotherapy using each agent.

This is the first report of PK interactions for the combination of an anti-EGF-R antibody and an anti-IGF-1R antibody. The co-administration of cetuximab and irinotecan with MK-0646 increased the MK-0646 AUC_0–168h_ by 25 %. As substantial pre-dose MK-0646 concentrations were observed before the co-administration treatment, MK-0646 accumulation contributed to the observed increase in MK-0646 exposure during that treatment, and the increase in exposure could not be attributed entirely to the potential interactions of the co-administered drugs. The co-administration of MK-0646 with cetuximab and irinotecan did not affect the PK of cetuximab and irinotecan. The GMR of the cetuximab AUC_0–168h_ (cetuximab + irinotecan + MK-0646/cetuximab + irinotecan) was 1.07, with a 90 % CI of (0.94, 1.21), which was within the bioequivalence bounds (0.80, 1.25). The co-administration of MK-0646 with cetuximab and irinotecan reduced the *C*_max_ and the AUC of SN-38. The SN-38 *C*_max_ decreased from 16.8 to 13.0 ng/mL upon MK-0646 co-administration, whereas the SN-38 AUC_0–24h_ decreased by 13 %, with a 90 % CI of (0.74, 1.01). The PK interaction between cetuximab and irinotecan was previously assessed by Delbaldo et al. [15]. The results of that study showed an apparent decrease in the SN-38 *C*_max_ and the AUC upon the co-administration of cetuximab and irinotecan; however, the difference was not statistically significant because of the large inter-subject variability, and the authors concluded that no PK interaction existed between the two drugs. The mean SN-38 concentration-time profiles of the current study resembled those reported by Delbaldo et al. Since the irinotecan dosing regimens (150 mg/m^2^/2 weeks in the current study vs. 350 mg/m^2^/3 weeks in the Delbaldo et al. study) differed, a quantitative comparison of the profiles is not feasible. Taken together, these observations suggest that the co-administration of MK-0646 with cetuximab and irinotecan had little effect, if any, on the PK of the three components of the combination.

The KRAS status has been reported to be a predictive marker of the response to cetuximab [16–18], but patients were enrolled in this study regardless of the KRAS status according to the Japanese package inserts for cetuximab at that time. Though the response rate was not the primary endpoint in this phase I study and was obtained from only a small number of patients, an add-on effect of MK-0646 was not observed; instead, the rate appeared to be lower (3/20; 15.0 %) than historical data reported for the combination of cetuximab and irinotecan (22.9 % [9], 30.8 % [19]). No clear difference in the response rate between patients with the KRAS wild-type and those with the mutant-type was seen in this study. A randomized phase II/III study of the triple combination was conducted in patients with chemorefractory metastatic colorectal cancer with a wild-type KRAS status [20]. This phase II/III study was stopped at the first interim analysis because the addition of MK-0646 to cetuximab and irinotecan worsened the progression-free survival and overall survival. A comprehensive molecular analysis was undertaken retrospectively to identify possible predictors of cetuximab resistance and MK-0646 response [21]. These data supported IGF-1 and IGF-2 as potential biomarkers for a response to MK-0646 therapy and a high IGF-1 level as a marker of resistance to cetuximab therapy. Further analyses in a molecularly selected population of metastatic colorectal cancer are underway.

A phase II study of IMC-A12, another anti-IGF-IR antibody, alone and in combination with cetuximab in patients with colorectal cancer refractory to EGFR inhibitor did not demonstrate meaningful anti-tumor activity [22]. Disappointing results have also been reported for other anti-IGF-1R antibodies in the treatment of non-small-cell lung cancer (NSCLC) [23, 24]. These study results underline the importance of identifying predictive biomarkers of IGF-1R dependence in the development of future anti-IGF-IR antibodies.

In conclusion, the triple combination of MK-0646, cetuximab, and irinotecan was well tolerated in Japanese patients with advanced colorectal cancer. The present results indicated a minimal potential for PK interactions between MK-0646 and cetuximab and between MK-0646 and irinotecan/SN-38.

## References

[1] Saltz LB, Minsky B (2002). Adjuvant therapy of cancers of the colon and rectum. Surg Clin North Am.

[2] Tournigand C, André T, Achille E, Lledo G, Flesh M, Mery-Mignard D (2004). FOLFIRI followed by FOLFOX6 or the reverse sequence in advanced colorectal cancer: a randomized GERCOR study. J Clin Oncol.

[3] Czito BG, Hong TJ, Cohen DP, Tyler DS, Lee CG, Anscher MS (2004). A phase I trial of preoperative eniluracil plus 5-fluorouracil and radiation for locally advanced or unresectable adenocarcinoma of the rectum and colon. Int J Radiat Oncol Biol Phys.

[4] Veronese ML, Sun W, Giantonio B, Berlin J, Shults J, Davis L (2005). A phase II trial of gefitinib with 5-fluorouracil, leucovorin, and irinotecan in patients with colorectal cancer. Br J Cancer.

[5] Cohen S (2004). Origins of growth factors: NGF and EGF. Ann NY Acad Sci.

[6] Resnick MB, Routhier J, Konkin T, Sabo E, Pricolo VE (2004). Epidermal growth factor receptor, c-MET, beta-catenin, and p53 expression as prognostic indicators in stage II colon cancer: a tissue microarray study. Clin Cancer Res.

[7] Galizia G, Lieto E, Ferraraccio F, De Vita F, Castellano P, Orditura M (2006). Prognostic significance of epidermal growth factor receptor expression in colon cancer patients undergoing curative surgery. Ann Surg Oncol.

[8] Yasui W, Sumiyoshi H, Hata J, Kameda T, Ochiai A, Ito H (1988). Expression of epidermal growth factor receptor in human gastric and colonic carcinomas. Cancer Res.

[9] Cunningham D, Humblet Y, Siena S, Khayat D, Bleiberg H, Santoro A (2004). Cetuximab monotherapy and cetuximab plus irinotecan in irinotecan-refractory metastatic colorectal cancer. N Engl J Med.

[10] Goetsch L, Gonzalez A, Leger O, Beck A, Pauwels PJ, Haeuw JF (2005). A recombinant humanized anti-insulin-like growth factor receptor type I antibody (h7C10) enhances the antitumor activity of vinorelbine and anti-epidermal growth factor receptor therapy against human cancer xenografts. Int J Cancer.

[11] Adams TE, McKern NM, Ward CW (2004). Signalling by the type 1 insulin-like growth factor receptor: interplay with the epidermal growth factor receptor. Growth Factors.

[12] Atzori F, Tabernero J, Cervantes A, Prudkin L, Andreu J, Rodríguez-Braun E (2011). A phase I pharmacokinetic and pharmacodynamic study of dalotuzumab (MK-0646), an anti-insulin-like growth factor-1 receptor monoclonal antibody, in patients with advanced solid tumors. Clin Cancer Res.

[13] Watkins DJ, Tabernero J, Schmoll HJ, Trarbach T, Ramos FJ, Hsu K et al (2009) A phase II study of the anti-IGFR antibody MK-0646 in combination with cetuximab and irinotecan in the treatment of chemorefractory metastatic colorectal cancer. J Clin Oncol 27(Supp 15):abstract 4127

[14] Weroha SJ, Haluska P (2008). IGF-1 receptor inhibitors in clinical trials—early lessons. J Mammary Gland Biol Neoplasia.

[15] Delbaldo C, Pierga JY, Dieras V, Faivre S, Laurence V, Vedovato JC (2005). Pharmacokinetic profile of cetuximab (Erbitux™) alone and in combination with irinotecan in patients with advanced EGFR-positive adenocarcinoma. Eur J Cancer.

[16] Lièvre A, Bachet JB, Le Corre D, Boige V, Landi B, Emile JF (2006). KRAS mutation status is predictive of response to cetuximab therapy in colorectal cancer. Cancer Res.

[17] Van Cutsem E, Köhne CH, Hitre E, Zaluski J, Chang Chien CR, Makhson A (2009). Cetuximab and chemotherapy as initial treatment for metastatic colorectal cancer. N Engl J Med.

[18] Karapetis CS, Khambata-Ford S, Jonker DJ, O’Callaghan CJ, Tu D, Tebbutt NC (2008). K-ras mutations and benefit from cetuximab in advanced colorectal cancer. N Engl J Med.

[19] Tahara M, Shirao K, Boku N, Yamaguchi K, Komatsu Y, Inaba Y (2008). Multicenter phase II study of cetuximab plus irinotecan in metastatic colorectal carcinoma refractory to irinotecan, oxaliplatin and fluoropyrimidines. Jpn J Clin Oncol.

[20] Watkins DJ, Tabernero J, Schmoll H, Trarbach T, Ramos FJ, Howe J et al (2011) A randomized phase II/III study of the anti-IGF-1R antibody MK-0646 (dalotuzumab) in combination with cetuximab (Cx) and irinotecan (Ir) in the treatment of chemorefractory metastatic colorectal cancer (mCRC) with wild-type (wt) KRAS status. J Clin Oncol 29(Suppl):abstract 3501

[21] Watkins DJ, Ayers M, Cunningham D, Tabernero J, Tejpar S, Kim TY et al (2012) Molecular analysis of the randomized phase II/III study of the anti-IGF-1R antibody dalotuzumab (MK-0646) in combination with cetuximab (Cx) and irinotecan (Ir) in the treatment of chemorefractory KRAS wild-type metastatic colorectal cancer (mCRC). J Clin Oncol 30(Suppl):abstract 3531

[22] Reidy DL, Vakiani E, Fakih MG, Saif MW, Hecht JR, Goodman-Davis N (2010). Randomized, phase II study of the insulin-like growth factor-1 receptor inhibitor IMC-A12, with or without cetuximab, in patients with cetuximab- or panitumumab-refractory metastatic colorectal cancer. J Clin Oncol.

[23] Ramalingam SS, Spigel DR, Chen D, Steins MB, Engelman JA, Schneider CP (2011). Randomized phase II study of erlotinib in combination with placebo or R1507, a monoclonal antibody to insulin-like growth factor-1 receptor, for advanced-stage non-small-cell lung cancer. J Clin Oncol.

[24] Jassem J, Langer CJ, Karp DD, Mok T, Benner RJ, Green SJ et al (2010) Randomized, open label, phase III trial of figitumumab in combination with paclitaxel and carboplatin versus paclitaxel and carboplatin in patients with non-small cell lung cancer (NSCLC). J Clin Oncol 28(Suppl 15):abstract 7500 10.1200/JCO.2013.54.4932 PMC4067944 24888810

